# Humanizing Miniature Hearts through 4-Flow Cannulation Perfusion Decellularization and Recellularization

**DOI:** 10.1038/s41598-018-25883-x

**Published:** 2018-05-10

**Authors:** Duong T. Nguyen, Matthew O’Hara, Cecilia Graneli, Ryan Hicks, Tasso Miliotis, Ann-Christin Nyström, Sara Hansson, Pia Davidsson, Li-Ming Gan, Maria Chiara Magnone, Magnus Althage, Sepideh Heydarkhan-Hagvall

**Affiliations:** 1 Cardiovascular, Renal and Metabolic Diseases, Innovative Medicines and Early Development Biotech Unit, AstraZeneca, Mölndal, Sweden; 2 School of Bioscience, Systems Biology Research Center, University of Skövde, Skövde, Sweden; 3 Discovery Sciences, Innovative Medicines and Early Development Biotech Unit, AstraZeneca, Mölndal, Sweden; 4 Early Clinical and Development, Innovative Medicines and Early Development Biotech Unit, AstraZeneca, Mölndal, Sweden

## Abstract

Despite improvements in pre-clinical drug testing models, predictability of clinical outcomes continues to be inadequate and costly due to poor evidence of drug metabolism. Humanized miniature organs integrating decellularized rodent organs with tissue specific cells are translational models that can provide further physiological understanding and evidence. Here, we evaluated 4-Flow cannulated rat hearts as the fundamental humanized organ model for cardiovascular drug validation. Results show clearance of cellular components in all chambers in 4-Flow hearts with efficient perfusion into both coronary arteries and cardiac veins. Furthermore, material characterization depicts preserved organization and content of important matrix proteins such as collagens, laminin, and elastin. With access to the complete vascular network, different human cell types were delivered to show spatial distribution and integration into the matrix under perfusion for up to three weeks. The feature of 4-Flow cannulation is the preservation of whole heart conformity enabling ventricular pacing via the pulmonary vein as demonstrated by noninvasive monitoring with fluid pressure and ultrasound imaging. Consequently, 4-Flow hearts surmounting organ mimicry challenges with intact complexity in vasculature and mechanical compliance of the whole organ providing an ideal platform for improving pre-clinical drug validation in addition to understanding cardiovascular diseases.

## Introduction

Even with advancements in technology, the drug discovery pipeline stagnates at a 5% success rate and an average 13-year timeline^1^. Hence, better predictability of molecular mechanism, specificity, and potency at the early stages of drug validation will increasing efficiency and alleviate economic strain in later stages. Translational science research strategies strive to establish highly relevant human based *in vivo* and *in vitro* models to improve prediction of targeted metabolism with minimal side effects^1–3^.

With cardiovascular diseases as the leading cause of death worldwide, the demand for better cardiovascular treatments and predictions of therapeutic outcomes is a substantial challenge for translational research. Therefore, models that can simulate the heart’s physiology and functionality during normal and disease conditions will provide a comprehensive understanding on drug metabolism^2,4,5^. The ideal model can predict at the cellular and tissue level the desired therapeutic effects on the disease in addition to the adverse effects imposed on healthy tissues. Engineered heart tissues constructed from synthetic and natural biomaterials are the most interesting modalities; however, all materials not derived from animal tissues requires fine tuning of composition, architecture, mechanical properties, and compatibility for partial mimickry of a complex organ^3,6,7^. Utilizing decellularized animal hearts will circumvent many engineering limitations and further reform the current translational strategy^1,3,6,8–11^. Elaborating from previous studies, three-dimensional platforms progress differentiation of human embryonic stem cells and human induced pluripotent stem iPS) cells towards cardiac myocytes exhibiting cell-to-cell interaction, matrix development and organization, and heterogeneous metabolism at the tissue level^6,7,12–15^. Others have shown the acellular heart can support the attachment of human iPS cells, cardiac fibroblasts, human embryonic stem cells, and iPS derived cardiac progenitor cells (iPS-CPCs)^13,14^. Consequently, a humanized heart as an *in vitro* model will elevate the quality and credibility of evidence during drug testing by understanding drug metabolism and interactions within a complex tissue^1,6,9–11^.

Current methods for harvesting rodent hearts for perfusion decellularization follow the Langendorff isolation method, intended for *ex vivo* experiments, by cannulating the ascending aorta and preserving aortic valve function to perfuse only through the coronary arteries^4,11,16,17^. The modified Langendorff method includes an invasive insertion of a balloon through the left atrium into the left ventricle for pacing with a consequence of removing the left atrium and inducing non-physiological exertion to the ventricle wall^4^. For *ex vivo* experiments, the Langendorff method is important to evaluate cardiac functionality in response to pharmacological treatments. However, for decellularization and recellularization purposes, the Langendorff cannulation has limitations hindering the development of the whole heart model^12,18,19^. Additional challenges arise during the recellularization process such as complete retention of total cell number, homogenous distribution of cells within the matrix, and clearance of the vascular network for continuous nutrient perfusion. Previously, repopulation via localized manual injections showed low cell retention, low reproducibility, additional matrix damage, and clustered distribution of the cells at unspecific locations^10,12^. Others have performed perfusion delivery of cells through the aorta cannulation without confirming aortic valve competence which can lead to cells being washed out easily, or accumulating in the ventricle chamber, or precluding the vascular network^12,15,17,19^. Hence, utilizing a perfusion system with integrated pressure monitoring and feedback will streamline the decellularization process and prevent high mechanical stress on the cells during perfusion recellularization^14,18^. Even when the cells are homogenously flowing through the vascular network during recellularization, dissipation of the cells into the tissue is an important aspect to optimize for vascular clearance and cell survival. However, in utilizing the vascular network to repopulate the vascular basement membrane will retain the cells within the vessels which is optimal for endothelial cell repopulation but not all other cardiac cells^15,20^.

Here, we have developed a method - termed 4-Flow cannulation - optimized for isolating hearts for decellularization and recellularization to maintain heart physiology with a complete circulation and organ conformity. With four different cannulations as perfusion inlets and outlets, decellularization reagents and recellularization solutions are delivered through the coronary arteries and cardiac veins. The versatility of the 4-Flow cannulation will be validated when compared to the current isolation method - Langendorff - which was adopted from *ex vivo* applications with only the aorta cannulation. Techniques such as resin casting will be used to map the perfusion network of the whole organ and multiphoton imaging will illustrate three dimensional architecture of the preserved matrix. In addition, various recellularization parameters such as cell types and culture conditions will be evaluated to demonstrate improved spatial distribution, survival, and integration within the acellular matrix. The key attribute is the capability to induce ventricle pacing emulating natural expansion through the left atrium and dynamic aortic valve. Hence, the objective is to highlight 4-Flow cannulated hearts as the humanized predictive model for translational research in cardiac drug development.

## Results

### Comparison of Decellularization and Vasculature

**Figure 1:**
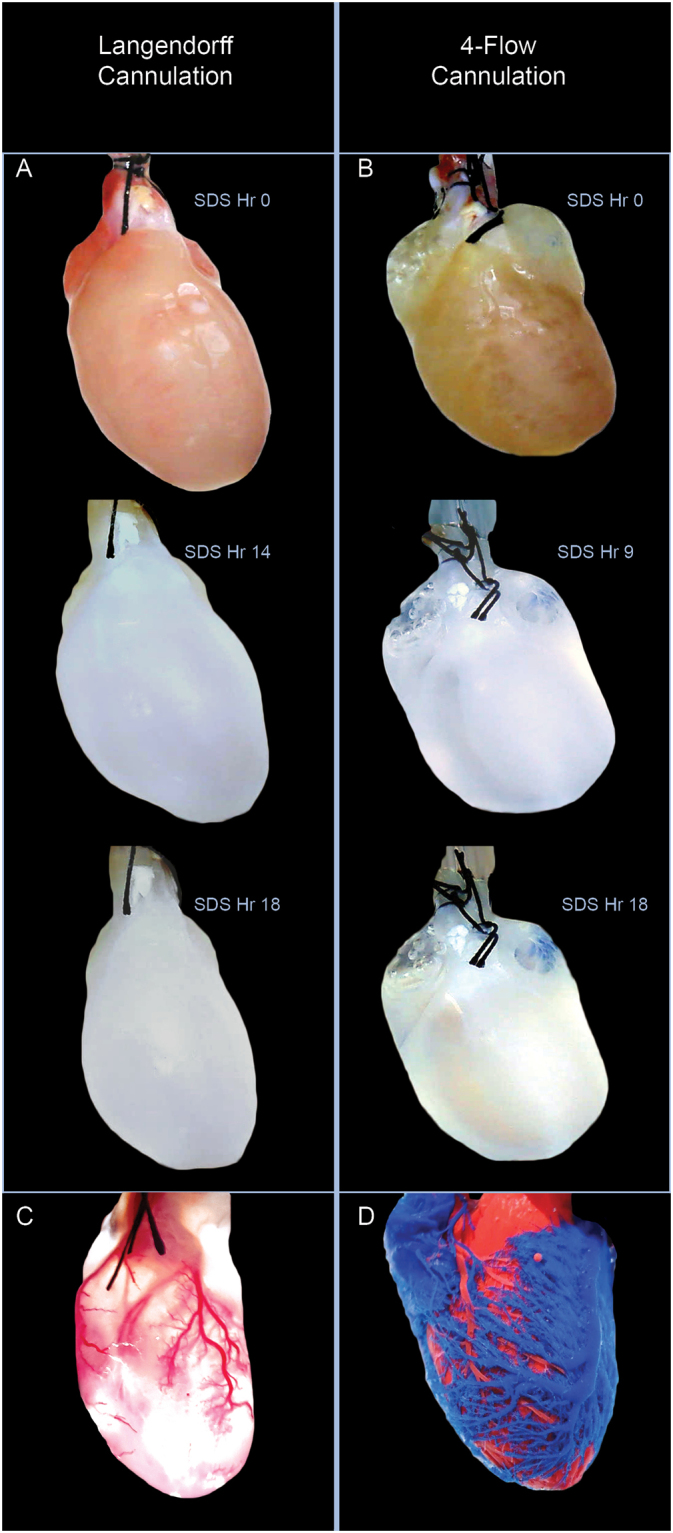
Macroscopic observation of differences between Langendorff and 4-Flow cannulated hearts decellularized and resin casted. (**A**,**B**) Time lapse comparison between Langendorff and 4-Flow cannulation perfused with 3 L of 1% sodium dodecyl sulfate solution under constant pressure. The 4-Flow cannulated hearts has the most clarity in all chambers of the heart. (**C**,**D**) Perfusion of the colored resin casting polymer illustrates the limited accessibility of the Langendorff cannulation as compared to the vascular interaction between coronary arteries and cardiac veins of the 4-Flow heart. The red colored polymeric solution was perfused through the AA for both the Langendorff and 4-Flow hearts while the blue colored polymeric solution was perfused through the SV of the 4-Flow heart. (**C**) The decellularized tissue was intentionally preserved for the Langendorff resin cast; however, (**D**) the tissue was removed in the 4-Flow heart. Images are representative of replicates (n = 4).

The vascular network and the decellularized matrix were characterized for both cannulation methods to demonstrate that 4-Flow hearts have the proper cannulations for producing a humanized miniature organ model as an essential pre-clinical model. The 4-Flow hearts were cannulated at the superior vena cava (SV), ascending aorta (AA), pulmonary artery (PA), and pulmonary vein (PV) while Langendorff hearts only have the AA cannulation. The time lapse images of the decellularization process after a heparin rinse illustrates the differences in heart shape and time of cell clearance of the two cannulation methods under constant pressure perfusion of three liters sodium dodecyl sulfate (SDS) (Fig. 1). Under perfusion decellularization with constant pressure, after nine hours, the 4-Flow hearts were visually clear of cell matter in all chambers in contrast to the opaque atrial tissue remaining in the Langendorff cannulated hearts at 14 hours (Fig. 1A,B). Both cannulation methods; however, took 18 hours to finish perfusing three liters of SDS (Fig. 1A,B). When decellularization was complete, the overall shape of the hearts with 4-Flow perfusion is more similar to native hearts with inflated atria and ventricles. In order to visualize the vasculature network and showcase the functional aortic valve, resin casting was performed for the decellularized Langendorff and 4-Flow hearts (Fig. 1C,D). Since the red resin cast flowed only into coronary arteries and not into the ventricle chamber, the function of the aortic valve for both cannulation methods was preserved after decellularization (Fig. 1C,D). The resin cast branched into the right ventricle, septum, and left ventricle from large vessels to capillaries (Fig. 1C). The 4-Flow heart casting depicts the branching and interaction of both coronary arteries in red resin and cardiac veins in blue resin on all chamber walls of the decellularized tissue. The coronary sinus and anterior cardiac veins can be seen casted in blue descending into smaller vessels partially forming the right and left ventricle wall (Fig. 1D).

### Evaluation of Matrix Content

**Figure 2:**
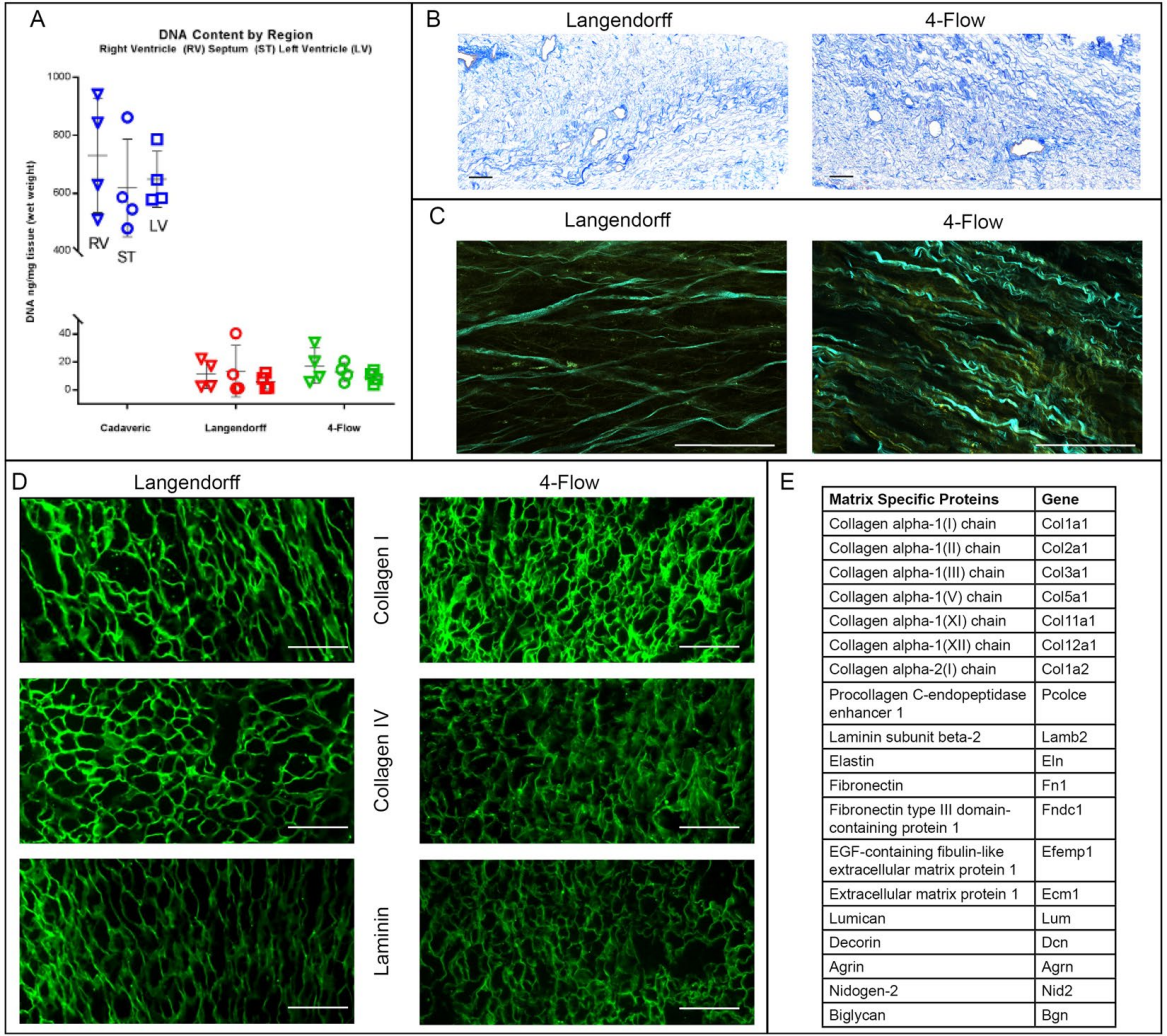
Characterization of decellularized heart matrix for DNA and extracellular matrix content with DNA quantification, histological staining, and multiphoton imaging. (**A**) DNA content of the right ventricle (RV, triangles), septum (ST, circles), and left ventricle (LV, squares) for cadaveric hearts compared to decellularized Langendorff (n = 4) or 4-Flow cannulated hearts (n = 4). (**B**) Masson’s trichrome staining of representative Langendorff (n = 4) and 4-Flow (n = 4) hearts for preserved collagen (blue) and clearance of nuclear remnants (absence of positively stained purple nuclei). (**C**) Multiphoton microscopy visualization of collagen (pseudocolored - cyan) and elastin (pseudocolored - yellow) fibrillar strcutures and organization in 3D (n = 4). (**D**) Immunostaining of histology sections important extracellular matrix proteins: collagen I, collagen IV, and laminin (n = 4). (**E**) The list of preserved extracellular matrix proteins in decellularized 4-Flow hearts as detected by mass spectrometry (n = 4).

**Figure 3:**
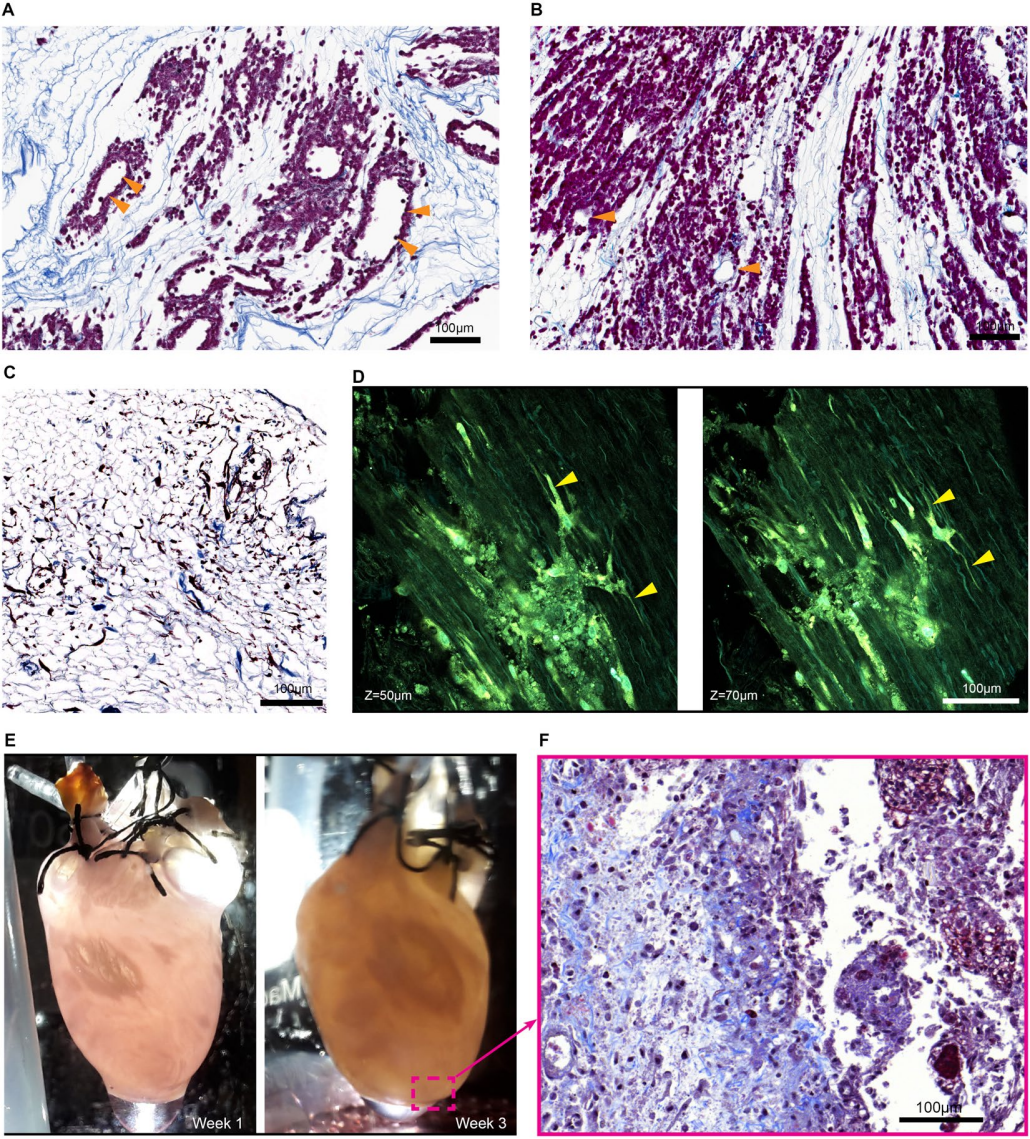
Perfusion recellularization of HEK293 cells, fibroblasts, and h-iPS-CPCs into 4-Flow cannulated hearts. (**A**) HEK293 cells delivered through only the SV of a 4-Flow heart perfuse through the cardiac veins and throughout the walls of the heart and distribute within the matrix proximal to the vasculature maintaining a hollow lumen (n = 3). (**B**) HEK293 cells delivered through both SV to the cardiac vein and AA to the coronary arteries are more thoroughly dispersed into the tissue walls leaving the vasculature hollow (orange triangles) (n = 3). (**C**) Perfusion culture of a primary human cardiac fibroblasts repopulated 4-Flow heart demonstrates cell survival, attachment, and invasion into the tissue (n = 3). Multiphoton imaging captures collagen fiber structures through second harmonic generation seen in cyan while autofluorescence of elastin and cells, and Hoechst nuclear staining is shown in green-yellow. (**D**) Multiphoton imaging of the 2-week 4-Flow heart reveals the lamellipodia extensions of the fibroblasts (yellow triangles) interacting with the decellularized matrix in three-dimension (n = 3). (**E**) Macroscopic observation of the recellularized heart with h-iPS-CPCs during the 3 weeks of culture indicate change in opacity (n = 3). (**F**) Corresponding representative image of Masson’s Trichrome staining shows cells are embedded within the collagen matrix. Histology sections were stained with Masson’s trichrome in which cell nuclei are purple and collagen fibers are blue.

DNA content of the decellularized tissues was characterized quantitatively and qualitatively demonstrating that perfusion with only SDS can remove all cellular components for both 4-Flow and Langendorff cannulated hearts (Fig. 2). In addition, various characterization methods of extracellular matrix (ECM) protein and organization indicate preservation of major matrix components for both cannulation methods (Fig. 2). Since the areas of interest are at the ventricle walls, DNA content of the right ventricle wall, septum, and left ventricle wall for both cannulation methods show less than 50 ng/mg DNA (Fig. 2A). Clearance of nuclear content from the tissues with Masson’s trichrome staining is evident in while preserving collagen organization around vascular structures (Fig. 2B). The 3D spatial organization of collagen and elastin fibers was demonstrated with multiphoton imaging (Fig. 3D). To specifically examine collagen I, collagen IV, and laminin, immunostain was performed and results illustrate preservation of these important ECM proteins. Furthermore, mass spectrometry analysis of protein extracts from 4-Flow decellularized tissues detected that 20 different extracellular matrix proteins were retained (Fig. 2E).

### Recellularization of 4-Flow Hearts

The advantage of the 4-Flow cannulation is highlighted with cell repopulation by perfusion through both SV and AA (Fig. 3). HEK293 cells were used for short term 24 hour recellularization investigation of cell distribution while cardiac fibroblasts were used for a 2-week investigation of cell attachment, invasion, and survival (Fig. 3A–D). Within 24 hours the HEK293 cells delivered through the SV were distributed throughout the decellularized organ and dispersed into the tissue leaving the vascular lumen clear for continuous perfusion of medium (Fig. 3A). With perfusion of HEK293 cells through both SV and AA, dense areas of widely dispersed cells are evident around cleared vascular structures (Fig. 3B). Primary human cardiac fibroblasts recellularization for 2 weeks were used to evaluate cell attachment, migration, and cell-matrix interaction which is evident through histology and multiphoton analysis (Fig. 3C,D). The fibroblasts’ elaborate lamellipodia extensions through the vessel walls and attachment to the ECM can be observed by multiphoton imaging (Fig. 3D). When the h-iPS-CPCs were perfused to repopulate the 4-Flow hearts, growth of cell patches can be observed macroscopically during 3 weeks of dynamic culture (Fig. 3E). Histology analysis reveals the integration of h-iPS-CPCs into the tissue as single cells surrounded by matrix structures (Fig. 3F).

### Mechanics of 4-Flow Hearts

**Figure 4:**
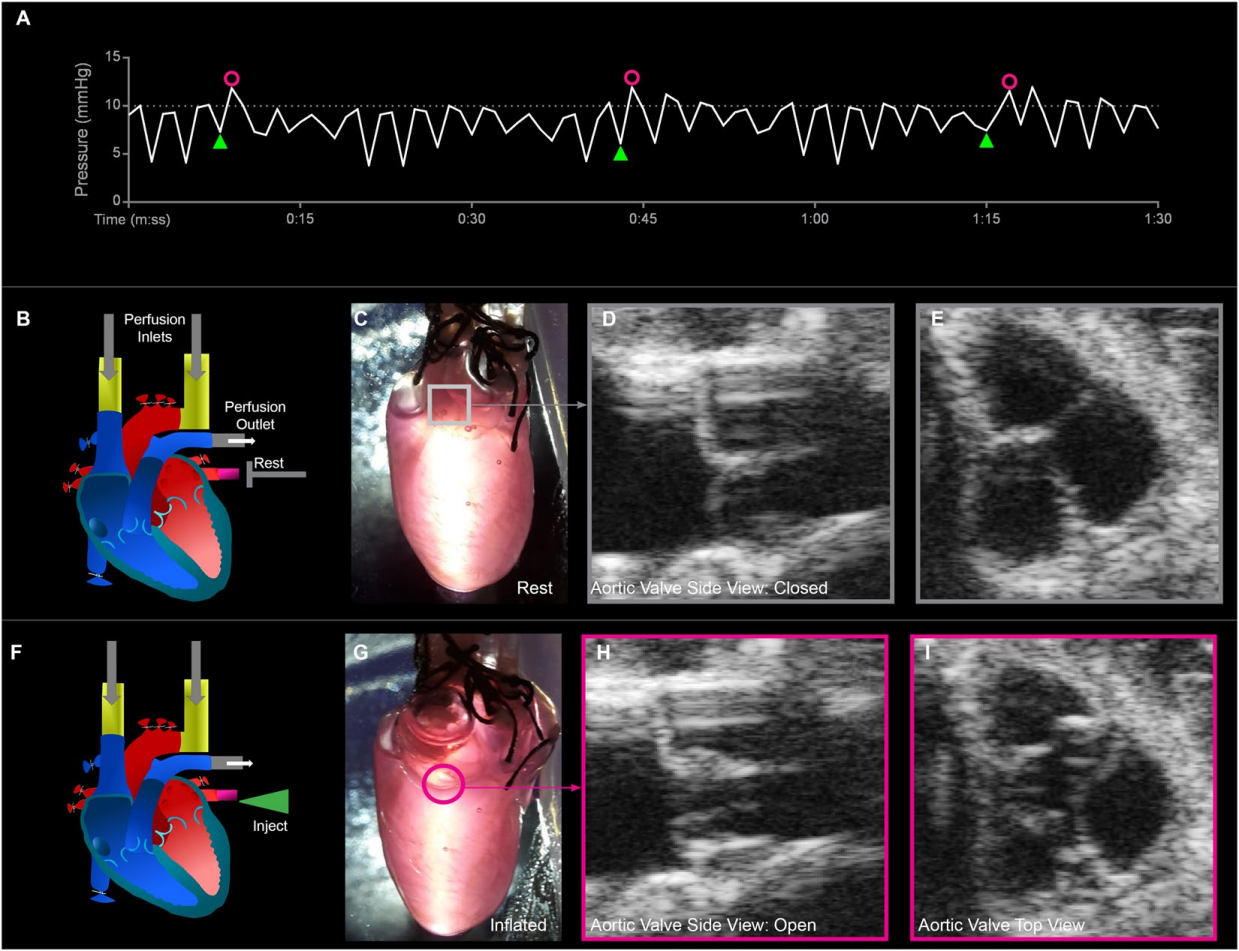
Observations of decellularized 4-Flow heart conformity and aortic valve competence under cyclic mechanical stimulation and constant perfusion (accellular heart). (**A**) By monitoring the fluid pressure fluctuations right before the ascending aorta, characteristic pressure fluxes (pink circles) due to PV injection (green triangles) can be distinguished from the relaxed pressure ranges. During rest with only SV and AA perfusion, the heart is at the relaxed phase with the aortic valve closed maintaining pressure ranging from 5–10 mmHg due to the peristaltic perfusion. During the injection phase initiated by the injection of medium (green triangle) through the PV into the left atrium and then to the left ventricle inflating and increasing pressure within a few seconds before the aortic valve opens (pink circle). Once the aortic valve opens, the medium is dissipated into the coronary arteries resulting in lowered ventricular pressure and closing of the aortic valve returning to the rest phase. (**B**,**F**) The schematic illustrates the injection route at the PV (rest: gray T, injection: green triangle), perfusion inlets at the AA and SV, flow outlet, and also the state of each valve. With cyclic injection of medium through the PV in addition to continuous perfusion through the SV and AA the heart undergoes a relaxed phase (**C**) and a stretched phase (**G**). (**C**,**D**,**H**,**I**) The cyclic ventricle inflation and deflation, and opening and closing of the aortic valve can be observed through ultrasound imaging.

Mechanical stimulation or ventricle pacing is the key feature of the 4-Flow cannulation achievable by preserving the left atrium via PV cannulation and the competence of the aortic valve after decellularization. Here, a decellularized 4-Flow heart underwent cyclic injections of medium into the PV to conduct ventricle pacing. The medium flows from the left atrium into the left ventricle chamber increasing fluid pressure which causes the aortic valve to open dissipate medium into the coronary circulation (Fig. 4). Conveniently, the continuous fluid pressure monitoring at the ascending aorta, integrated in the perfusion system, is an automated method to confirm PV injection illustrated by the flux in pressure to 12 mmHg and aortic valve mechanical function indicated by the fall in pressure to the relaxed pressure range of 5–10 mmHg (Fig. 4A,B,F). When the aortic valve is open, the pressure is relieved until the pressure from AA perfusion closes the valve (4F-4I, 4E-4E, respectively).The heart inflation can be observed (Fig. 4C,G) macroscopically while aortic valve competence was confirmed by ultrasound imaging (Video 1, Fig. 4D,E,H,I). During long-term culture, only the pressure reading is necessary to confirm aortic valve function.

## Discussion

With complex features such as a complete vascular network and a noninvasive route for ventricle pacing, 4-Flow hearts are ideal for decellularizing and repopulating with human cells as the physiological organ model to further understanding of cardiovascular diseases and related therapeutics. Previous investigations of whole heart models focus on optimizing decellularization protocols using different detergents and reagents, and improving repopulation via injections or perfusion with different cells types^9,13–15,17,20^. An important aspect that has been previously overlooked is the retention of the full circulation network of the heart with the proper cannulation for both decellularization and recellularization. Here, we showed that 4-Flow cannulated hearts are optimized for accessibility of the complete vascular network and conservation of whole heart mechanical conformity.

By accessing and controlling the perfusion into a heart via 4-Flow, decellularization is more efficient as compared to the Langendorff hearts with only access through the aorta. Furthermore, monitoring and controlling the fluid pressure right before the aortic valve ensures efficient and proper distribution of perfusates. The 4-flow cannulations has a perfusion pathway into the coronary arteries and cardiac veins as demonstrated by resin casting. The flow of the resin cast confirms the functionality of the aortic valve which is important to effectively deliver the cells through the coronary arteries and into the tissues rather than into the left ventricle chamber. In addition, utilizing the resin to visualize the perfusion pathway rather than dyes distinguishes fluid perfusion from diffusion^15,17^. Perfusion into both AA and SV directs flow inlet to coronary arteries and cardiac veins and flow outlets through PA and PV during decellularization and recellularization. Furthermore, utilizing perfusion at constant physiological pressure of 75 mmHg, the flow rate fluctuates to accommodate differences between each heart during decellularization^4^. Consequently, effective and proper decellularization can be completed by using SDS alone without the need for additional detergents^1,3,6,9–11^. Decellularized tissues were cleared of cellular components in addition to retaining important extracellular matrix proteins such as different collagens, elastin, and laminin for cell attachment and agrin a specific vascular protein.

One recellularization method utilizes injections of cells throughout the tissue which results in high loss of cells, localized distribution at unknown depths, and damage to the delicate tissue matrix^10,12^. Others have perfused cells through the AA to deliver cells into the coronary arteries; however, without monitoring the fluid pressure it is difficult to confirm the closure of the aortic valve and consequent flow into the arteries^13–15,17,19,20^. With 4-Flow under constant pressure monitoring, perfused cells are delivered with increased vascular coverage through the cardiac veins and coronary arteries with high confidence. Furthermore, distribution of the cells from the vascular network into the tissue is enhanced by increased vascular permeability. The short-term experiments with HEK293 cells demonstrated cell delivery through the SV and AA in which cells distribute throughout the matrix tissue in all chamber walls leaving the vascular lumen clear for undisturbed perfusion flow. Confirming clearance of vasculature ensures transport of medium to all populated areas within the heart. The repopulation with human primary cardiac fibroblasts provides evidence that primary cells have an affinity to the matrix to distribute, proliferate, and migrate under continuous dynamic perfusion. In h-iPS-CPC recellularization, 3 weeks of perfusion culture showed cell dispersion into the matrix and integration aligning with the orientation of the matrix fibers.

To summarize, this work has demonstrated that preserving the vascular accessibility with the 4-Flow cannulation improves whole organ perfusion reducing the amount of time needed for decellularization. Furthermore, the importance of maintaining and monitoring the competence of the aortic valve was established as a critical parameter for perfusion decellularization and recellularization. The Langendorff method is important for *ex vivo* experimentation; however, in decellularization and recellularization applications a more optimized cannulation method such as 4-Flow will improve perfusion of detergents and cells. By producing a miniature humanized heart model such as a 4-Flow heart, preservation of the physical and biological stimuli will promote progenitor cells towards mature and functional cells^8,21–23^. In addition, primary isolated patient cells and genetically modified progenitor cells can be used to generate patient centric models and disease models in which no translatable animal models exist. In conclusion, humanized miniature hearts can be an essential translational strategy to facilitate effective drug discovery in addition to understanding cardiac biology and diseases.

## Methods

### Animals

Animal care and experiments were carried out in compliance with Swedish national legislation and AstraZeneca Bioethics policies in accredited facilities (ethics approval number 104–2016 from Jordbruksverket). The project protocol was approved by the institutional oversight body, Djurskyddsorgan, as well as by regional Laboratory Animal Ethics Committee of Gothenburg as required by Swedish regulations.

### Perfusion Decellularization

Hearts were explanted from male Sprague-Dawley rats (250–300 g) under deep anesthesia with systemic heparin (2500 U/mL). The heart was explanted by cannulating the ascending aorta (AA), superior vena cava (SV), pulmonary artery (PA), and pulmonary vein (PV) while ligating all other inlets and outlets - termed 4-Flow cannulation - to maintain the complete and enclosed circulation. Polyethylene tubings (Intramedic Clay Adams Brand) were used to cannulate and secured with sutures. The AA and SV are perfusion inlets for flow through the coronary artery and cardiac vein, respectively, of decellularization reagents and recellularization solutions. The PA and PV are outlets for used reagents to exit. Hearts were stored in a 150 U/mL heparin phosphate buffered saline (PBS) solution in −80 °C until decellularization. All decellularization and recellularization were performed using a perfusion system (IVIVA Medical Inc., USA) controlled by constant flow or constant pressure in which fluid pressure right before the aortic valve is continuously monitored to ensure the closure of the valve for efficient delivery of all solutions through the coronary arteries. Hearts were allowed to equilibrate to room temperature before perfusion decellularization through the AA and SV at constant 75 mmHg of pressure (unless otherwise indicated) within the normal 70–130 mmHg pressure in a rat heart^4^. Sequentially, under sterile conditions and constant pressure monitoring, the heart was perfused with heparin (150 U/mL) PBS solution (up to 50 mL), 1% sodium dodecyl sulfate solution (in deionized water, 75 mmHg, 3 L), PBS with 1% antimycotic and antibiotic (up to 3 L), endonuclease in Hank’s balanced salt solution (25 U/mL, constant flow 2 mL/min, 1 hour), ultra-pure water wash (3 mL/min, 50 mL), collagenase IV solution (25 U/mL, 2 mL/min, 30 mL), ultra-pure water wash (3 mL/min, 50 mL), PBS with 1% antimycotic and antibiotic (3 mL/min, 500 mL). To prepare for recellularization, the heart was rinsed and primed with cell specific medium into custom made glass bottles (500 mL Duran Schott) with GL45 caps or modified GL45 lids with 3 outlets for perfusion tubings.

### Resin Casting

Batson No. 17 Anatomical Corrosion Kit (PolySciences Inc.) was used to demonstrate the perfusion pathways for decellularized 4-Flow and Langendorff cannulated hearts. The manufacturer’s protocol was optimized to produce 10 mL of resin solution. Briefly, 10 mL of the base solution was mixed with a droplet of pigment (red or blue) by manual stirring. Then 1 mL of the catalyst and 2 drops of the promoter were added sequentially and mixed with manual stirring. Once the solution is homogeneous, it was transferred to a 2 mL syringe for perfusion via syringe pump at a rate of 20 *μ*L/min into the suspended heart. The resin casted heart was allowed to cure overnight submerged in water. The 4-Flow casted heart was further treated to remove the tissue by submerging the heart in alternating 5% (w/v) potassium hydroxide and water in a 50 °C water bath. The decellularized tissue of the casted Langendorff heart was intentionally preserved in order to maintain the integrity of the detailed branching of the coronary arteries.

### DNA content

Freshly decellularized hearts were cut into region specific pieces and froze −80 °C directly for DNA extraction and quantification. DNA content was analyzed following the Quant-iT PicoGreen assay (Thermo Fisher) from extracted DNA obtained with the DNeasy Blood & Tissue Kit (Qiagen). Briefly, hearts were sectioned into the right ventricle, septum, and left ventricle (atria were excluded) and wet tissue weight was obtained. The section tissues were then digested for matrix characterization and DNA content according to the manufacturers’ protocols. Significant differences in DNA content were computed with two-way ANOVA using GraphPad Prism.

### Extracellular Matrix Characterization

Characterization of ECM content was conducted utilizing liquid chromatography tandem mass spectrometry, ECM assays (BioColor) and immunostainig of histology sections. Decellularized tissues from 4-Flow hearts were homogenized in a lysis buffer (2% SDS, 50 mM TEAB pH 8.5, 0.1 M DTT) and protease and phosphatase inhibitor cocktail (MS-SAFE) using the Precellys (Bertin Technologies) homogenization system. Protein extractions were processed to obtain tryptic peptides by using the filter aided sample preparation (FASP) method. Subsequently, tryptic peptides were quantified, TMT labelled, and dried. The dried samples were redissolved in 0.1% TFA and was subsequently fractionated into 8 fractions using a Pierce High pH Reversed-Phase Peptide Fractionation Kit, (Thermo Scientific). Samples were analyzed by nano Liquid Chromatography-tandem mass spectrometry (LC-MS/MS) (Easy nLC 1000, Proxeon) combined with Orbitrap Q Exactive mass spectrometer (Thermo Scientific) using a 2 hour gradient. The separation was performed using an Acclaim PepMap precolumn (75 *μ*M ID by 20 mm) connected to a 75 *μ*M by 500 mm analytical Easy Spray PepMap RSLC C18 column (2 *μ*m particles, 100 Å pore size; Thermo Scientific). The MS/MS spectra from each nano-LC-MS/MS fraction were searched against a selected rat tissue database (Swissprot) using the Proteome Discoverer 2.1 software (Thermo Scientific). The search criteria were as follows: tryptic digestion, one missed cleavage, carbamidomethylation (C) and TMT plex (K and N-terminal) were fixed modifications, the oxidation (M) as a variable modification, precursor ion mass tolerances were set at 6 ppm, and the fragment ion mass tolerance at 20 mmu. From the list of matched proteins, selected extracellular matrix proteins including proteoglycans, glycoproteins, and collagens were presented. For histological analysis, decellularized hearts were perfused with 4% paraformaldehyde to fixate the tissue then the samples were sent to a core facility (HistoCenter, Sweden) for paraffin embedding, sectioning at 4–8 *μ*m thick slices, and stained with Hemotoxylin and Eosin and Masson’s trichrome. Immunostaining specifically for collagen I (anti-collagen I, Novus, NB600-408), collagen IV (anti-collagen IV, AbCam, Ab6586), and laminin (anti-laminin, AbCam, Ab11575) were performed on sections after removing the paraffin. Sections were then incubated in a blocking solution (1% bovine serum albumin + 2% goat serum + 22.52 mg/mL glycine + 0.075% Triton-X + 0.01% Tween-20 in phosphate buffered saline (PBS)) for 30 minutes at room temperature. Primary antibody in the blocking solution was added and incubated for 1 hour at room temperature. Sections were washed with 0.1% Tween-20 in PBS three times for 5 minutes each time before incubating with secondary antibody (AlexaFluor 488 donkey anti-rabbit, Life Technologies). Coverslips were mounted onto the sections using ProLong Gold with DAPI (Molecular Probes).

### Cell Culture

Three different cell types were used in this study to demonstrate different properties of recellularization. For short 24 hour studies, mammalian stable cells, HEK293 (ATCC) were used and cultured in DMEM (1% antimycotic-antibiotic, 10% fetal bovine serum). HEK293 are a stable cell line that is commonly used in high-throughput screening of during initial drug screening^2,7^. Hence, HEK293 cells were used for initial toxicity testing of the matrix before continuing to more relevant human cells. The primary human cardiac fibroblasts (PromoCells) cultured in DMEM (1% antimycotic-antibiotic, 10% fetal bovine serum) were used to demonstrate matrix compatibility after 2 weeks of culture. Since the fibroblasts had minimal proliferation in 2D culture, only a small population was obtained for repopulating the heart. Human induced pluripotent stem (h-iPS) cells are produced and validated as previously described^24^. These iPS cells were differentiated to cardiac progenitor cells by a differentiation protocol adapted from Lian *et al*.^25^. Briefly, the h-iPS cells were seeded on 24-well plates coated with growth factor reduced matrigel at a density of 0.47 million cells per centimeter square in DEF-CS medium (Takara Bio) following the manufacturer’s protocol. The h-iPS cells were cultured for 2 additional days with DEF-CS medium before differentiation was initiated. On day 0 the medium was changed to RPMI (1649 Glutamax) supplemented with B-27 (minus insulin) and CHIR (12 *μ*M) induction was initiated and maintained for 24 hours. On day 2 IWP-2 (5 *μ*M) was added and maintained for 2 days. At day 5 The h-iPS-CPCs were used for recellularization experiments in RPMI (1649 Glutamax) medium with B27 (minus insulin) and on day 6 the medium was changed to RPMI (1649 Glutamax) supplemented with B-27 (with insulin). Spontaneous beating could be observed from day 10 of the differentiation in the control continuously cultured on plates, confirming presence of cardiomyocytes in the culture.

### Recellularization of decellularized heart

Before recellularization, the heart was rinsed with basal medium with recirculating perfusion of 20–30 mL of medium overnight in the incubator at 37 °C and 5% carbon dioxide with continuous monitoring of fluid pressure at the ascending aorta to ensure the valve is functional and closed. Afterwards, the heart was primed with 20–30 mL of complete medium with recirculating perfusion overnight. All recellularized hearts were cultured at 37 °C with 5% carbon dioxide and medium was changed 3 times a week. The following recellularization experiments were performed with a focus on cell distribution, attachment, and migration. Perfusion of 30 million HEK293 cells into each inlet (SV only, SV and AA; n = 3) was conducted at a rate of 0.5 mL/min to recirculate a cell suspension volume of 10 mL with DMEM. After 24 hours, perfusion of 4% para-formaldehyde was conducted to fix the hearts. Perfusion of cardiac fibroblasts at 0.3 million cells through each inlet (SV and AA) by recirculating a total volume of 10 mL with DMEM and constant rate 0.5 mL/min (n = 3). After 2 weeks, perfusion of 4% para-formaldehyde was conducted to fix the hearts for histology sectioning and multiphoton imaging. The h-iPS-CPCs at day 5 were used for recellularization experiments in RPMI (1649 Glutamax) medium supplemented with B27 (minus insulin) (n = 3). For h-iPS-CPC recellularization, 3 rounds of cell seeding with a two hour gap were conducted to prevent cell aggregation. The first round of cell seeding with 15 mL of cell solution containing approximately 20 million cells were recirculated at 0.5 mL/min. After two hours 10 mL of the recirculated solution was removed. The second round of cell seeding with 10 mL of cell solution containing approximately 20 million cells were recirculated at 0.5 mL/min. The third round of cell seeding with an additional 10 mL of cell solution containing approximately 20 million cells were recirculated at 0.5 mL/min. The total amount of recirculating solution after three rounds of cell seeding was 25 mL which was allowed to recirculate until the next day. The medium was changed to RPMI Glutamax cont B27 with insulin the day after cell seeding and 3 times a week for the next 3 weeks. Samples were fixed by perfusing of 4% para-formaldehyde for histology sectioning.

### Mechanical Stimulation

Mechanical ventricle pacing was performed using a programmable syringe pump (Aladdin) in addition to the continuous perfusion of a decellularized heart (not repopulated with cells). Continuous injection and withdraw of 450 *μ*L at a rate of 3.5 mL/min and 1 mL/min, respectively, was optimized for maintaining aortic valve opening and closing. Perfusion through both SV and AA were necessary to close the aortic valve. The mechanical pacing can be monitored by the pressure gauge right before the aortic valve.The opening and closing of the aortic valve was visualized live with ultrasound imaging (video 1).

### Microscopy and Ultrasound Imaging

Histology slides were imaged using Aperio ScanScope and analyzed using ImageScope software. Fluorescent labeled immunostained slides were taken with a Nikon microscope. In consideration to the 3D and clarified nature of the samples, advanced multiphoton microscopy, at a core facility (Center for Cellular Imaging, Sweden), was utilized to perform volume imaging using a LSM 710 NLO Zeiss microscope coupled to a Mai Tai Deepsee or InSight laser (Spectra-Physics) with integrated pump laser tuned at 860 nm^26^. Multiphoton-excited autofluorescence and second harmonic generation (SHG) is advantageous for volumetric imaging to investigate cell-cell and cell-matrix interactions^27,28^. The fine structures and organization of elastin is obtained with autofluorescence while the anisotropic collagen fibers generate SHG signals. Due to the limited autofluorescence of the cardiac fibroblasts, Hoechst staining was used to highlight the cells. The success of 4-Flow hearts is the ventricle pacing and competence of the aortic valve which can be visualized noninvasively and under sterile conditions with ultrasound imaging was performed using the FujiFilm VisualSonics VEVO 2100 with the 40 MHz probe. Images were taken at 20 frames per second and compiled into a video. Still images of the ultrasound video were analyzed using ImageJ.
